# Nanomaterials for Remediation of Environmental Pollutants

**DOI:** 10.1155/2021/1764647

**Published:** 2021-12-28

**Authors:** Arpita Roy, Apoorva Sharma, Saanya Yadav, Leta Tesfaye Jule, Ramaswamy Krishnaraj

**Affiliations:** ^1^Department of Biotechnology, School of Engineering & Technology, Sharda University, Greater Noida, India; ^2^Department of Biotechnology, Delhi Technological University, Delhi, India; ^3^Centre for Excellence-Indigenous Knowledge, Innovative Technology Transfer and Entrepreneurship, Dambi Dollo University, Dembi Dolo, Ethiopia; ^4^Department of Physics, College of Natural and Computational Science, Dambi Dollo University, Dembi Dolo, Ethiopia; ^5^Department of Mechanical Engineering, Dambi Dollo University, Dembi Dolo, Ethiopia

## Abstract

Today, environmental contamination is a big concern for both developing and developed countries. The primary sources of contamination of land, water, and air are extensive industrialization and intense agricultural activities. Various traditional methods are available for the treatment of different pollutants in the environment, but all have some limitations. Due to this, an alternative method is required which is effective and less toxic and provides better outcomes. Nanomaterials have attracted a lot of interest in terms of environmental remediation. Because of their huge surface area and related high reactivity, nanomaterials perform better in environmental clean-up than other conventional approaches. They can be modified for specific uses to provide novel features. Due to the large surface-area-to-volume ratio and the presence of a larger number of reactive sites, nanoscale materials can be extremely reactive. These characteristics allow for higher interaction with contaminants, leading to a quick reduction of contaminant concentration. In the present review, an overview of different nanomaterials that are potential in the remediation of environmental pollutants has been discussed.

## 1. Introduction

The world is on the edge of a major environmental calamity that will cost us our fortune. The current state of the present environment is forever deteriorating. Environmental issues are piling up across the globe, and we have to behave as in an emergency on our planet. We have to gain a new perspective and approach calamities beforehand with new concepts and strategies and with our full awareness and seriousness. Pollution in nature, such as in air, water, and soil, undergoes millions of years to eradicate. Industry and automobile exhaust emissions are the main contributing factors for most of the environmental pollution [1].

Air pollutants like NO(x), SO_2_, highly reactive and toxic organic compounds, POPs like dioxins, and PAH (polycyclic aromatic hydrocarbons) are all hazardous for mankind [2]. When inhaled in high quantities, carbon monoxide (CO) can harm by immediate poisoning. When certain heavy metals such as Pb are taken into a living organism, they can vandalize by either immediate poisoning or chronic poisoning, depending on the different levels of exposure [3].

Pulmonary issues such as COPD, asthma, and bronchiolitis, cancers, heart diseases, CNS dysfunctions, and skin disorders are among the ailments caused by the aforementioned chemicals [4]. For ages, methods like condensation, flocculation, froth floatation, sand filtration, and AC adsorption are being widely used. However, these have their own limitations, such as inefficiency in scarping metal ions, high-energy input, and production of nonreusable chemicals. To overcome these problems, nanotechnology seems like an emerging option [5].

Nanotechnology possesses a prospect to significantly bestow in the development of cleaner, greener technologies that have major environmental and health advantages [6]. Nanotechnology techniques are being investigated for their potential to provide solutions for pollution management and mitigation, as well as to improve the performance of traditional environmental clean-up methods [7]. Nanotechnology can help the environment by reducing energy uptake during the manufacturing and production pathways, allowing products to get recycled after their usage, and developing and using environmentally friendly materials [8]. Nanotechnology presently holds great promise for addressing sustainability issues, but at the same time, we should also take into account the harm to the environment and human health [9]. Therefore, in this review, the importance of various nanomaterials that are capable of removing pollutants has been discussed.

## 2. Different Types of Pollutants

**Figure 1:**
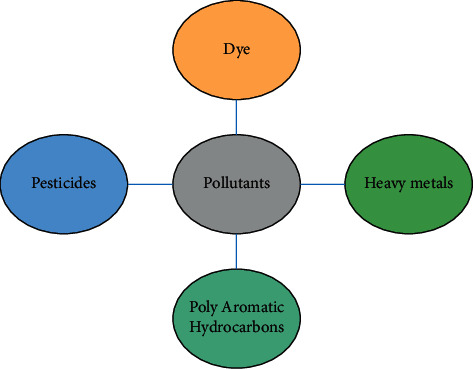
Types of pollutants.

Pollutants are particles that cause damage to the environment by polluting them. When exposed to these pollutants, life can be damaged, and consequences on humans and other organisms are well recognized. Pollutants can get into the environment in a number of different ways, both naturally and through humankind [10]. The different kinds of pollutants include dyes, heavy metals, pesticides, and poly aromatic hydrocarbons. (Figure 1).

### 2.1. Dyes

Dyes are a type of synthetic organic chemical that is used in a variety of industries, including textiles. As a result, during their manufacture and later during fabric dyeing, they have become frequent industrial environmental contaminants [11]. Synthetic dyes in textile wastewater decrease light penetration into rivers, altering the photosynthetic efficiency of aquatic vegetation and, as a result, the food supply of aquatic creatures. The thin coating of released dyes that might build on the receiving waters' surfaces reduces the quantity of dissolved oxygen, which has a serious impact on aquatic life. Biochemical oxygen demand (BOD) is increased by dye-containing industrial effluents. Dyes are basically persistent organic pollutants (POPs) that exist in the environment, and it has been a concern that these man-made chemicals are xenobiotic [12]. Dyes like Azure-B if introduced can affect the helical structure of an organism's DNA and duplex RNA. Disperse Red 1 and Disperse Orange 1 dyes are mutagenic in nature. Dyes such as Sudan I Dye (now banned), Basic Red 9 Dye, and Crystal Violet dye (induces tumor in fish) are carcinogenic in nature [13].

### 2.2. Heavy Metals

Heavy metal pollution is becoming more of an issue and a source of concern owing to the negative consequences it is causing all over the world [14]. Because of the rapidly increasing agriculture and metallurgy industries, inappropriate waste management, fertilizers, and pesticides, these come under inorganic pollutants and are being dumped in our waterways, land (soil), and the air (environment). Some of the most common heavy metals causing pollution are titanium (Ti)), iron (Fe), vanadium (V), chromium (Cr), manganese (Mn), cobalt (Co), copper (Cu), zinc (Zn), arsenic (As), molybdenum (Mo), nickel (Ni), cadmium (Cd), silver (Ag), tin (Sn), platinum (Pt), gold (Au), mercury (Hg), and lead (Pb) [14]. Heavy metals are classified as such because of their high gram atomic weight or higher densities. Heavy metals are now used to refer to poisonous synthetic metallic atoms or molecules and metalloids that are harmful to the environment and humans [15]. These toxic heavy metals are naturally available on the Earth's surface in their native state. The massive growth in their usage has resulted in an imminent influx of these metallic compounds in both the land and water natural resources. Heavy metal pollution has become an important component of anthropogenic activity, primarily due to metal mining, steelmaking, activities such as foundries and other metal-dependent industries, as well as metal leaching for extraction from various sources such as dumped landfills, excretion, domesticated poultry and chicken manure fertilizers, water runoffs, automobile industry, and road repair [16].

### 2.3. Pesticides

Pesticides are employed in agriculture for decades to safeguard crops and animals against insect infestations and to prevent the decrease in production. Pesticides are chemicals that are used to destroy unwanted organisms in gardens, agricultural farms, and other public locations. Pesticides, despite their use, may represent a threat to food safety, the environment, and all living things [17]. According to a survey conducted by the World Health Organization (WHO), around 1 million people are acutely poisoned as a result of pesticide exposure. Pesticides have a role in a variety of organic micropollutants with harmful environmental consequences. Biomagnification and bioconcentration are the two primary pollution processes [18]. Major interactions (receptor) in the human body for many pesticides are adipose fat tissue, causing these toxins to gather in lipids. DDT (dichlorodiphenyltrichloroethane) is an example of such a pesticide. If any human ingests any aquatic animal that has taken DDT with any food, it accumulates in the human body as DDT is lipophilic and dissolves in adipose tissues [19]. Inhibition or inability to reproduce, hormonal system disturbances, suppression of the immune system, animal and fish tumors death, malignancies, effects on intergenerational impacts of teratogens, DNA, cell damage, and significant muck on fish gills and crusts, indicative of poor fish health, and other physiological impacts including the weakening of the eggshell are some of the problems caused by pesticides pollutants [20].

### 2.4. Poly Aromatic Hydrocarbons

Due to their toxic, mutagenic, and carcinogenic potential, poly aromatic hydrocarbons are a category of persistent and widespread environmental contaminants that are also hazardous to people. With the rise in demand for petroleum goods, their emissions have grown. Contributors include combustion in lack of oxygen of organic products, for example, coal and firewood [21]. Thermally, poly aromatic hydrocarbons are extremely stable and resistant to decomposition. The melting and boiling temperatures of these aromatic compounds are quite high. Poly aromatic hydrocarbons with a lower molecular weight are typically water soluble, but their hydrophobicity and aqueous insolubility increase with each additional ring [22]. The US Environmental Protection Agency has identified naphthalene, phenanthrene (C_4_H_10_), anthracene (C_6_H_4_CH)_2_, fluoranthene (C_16_H_10_), pyrene (C_16_H_10_), chrysene (C_18_H_12_), benzo anthracene (C_18_H_12_), and benzo fluoranthene (C_20_H_12_) as pollutants that need to be kept in low concentration by constant monitoring.

## 3. Environmental and Health Consequences of Pollutants

Trace pollutants have been detected and analyzed during the recent decades with the establishment of refined techniques. However, subsequently with that emerging environmental concerns are to be reasoned with. The toxicological information regarding the majority of chemicals used is incomplete; long-term usage information or low-level exposure still lacks making the environmental challenges hidden. The path for identification of various sustainable as well as long-lasting techniques to counterattack the challenges of new emerging and identified pollutants with a detailed study of their consequences will be fruitful for human generations.

### 3.1. Environmental Consequences

#### 3.1.1. Aquatic

One of the largest sources of aquatic pollution by environmental pollutants is Cultural Eutrophication. The increased rate of food production and population size has resulted in the discharge of nitrogen and phosphorus-rich compounds in water bodies [23]. This in turn has led to the expansion of harmful algal blooms toxic to the native aquatic plant and animal species and dysregulation of the biogeochemical cycle of persistently present organic pollutants in the natural environment [24]. Eutrophication has undesirably caused restriction of water use by fisheries, industries, and consumption as potable water because of decreased oxygen percentages and high ratios of poisonous compounds [25]. Another problem characterized by persistent organic pollutants is biomagnification, that is, the accumulation of poorly metabolizable, moderate to highly hydrophobic substances that are circulated in food webs [26]. The major effect caused due to these is a significant reduction in the numbers of biologically important local and native species which play an important role in the ecosystem.

The anthropogenic pollutants have threatened marine integrity; elevated carbon dioxide and trace metal levels have caused the imbalance of acid base homeostasis in organisms, thereby affecting their chance of survival and performance often also called Ocean Acidification [27]. It has caused an untimely calcification of coral reefs, microorganisms like zooplanktons, and shellfishes [28]. The aquatic toxicology reveals the presence of mixed toxicities including heavy metals [29] and persistent organic pollutants (POPs), including perfluoro alkylated acids (PFAS) worldwide [30]. Water is one of the most vulnerable and essential components of the environment and its pollution is a matter of great concern [31].

#### 3.1.2. Terrestrial

The major contributors to environmental pollution are industries. The unauthorized dumping of pollutants such as aromatic hydrocarbons, plasticizers, and various xenobiotic compounds is a threat to the terrestrial ecosystem [32]. They are holders of mutagenic and carcinogenic properties and have decreased agricultural land production due to the accumulation of toxic compounds that have an impact on seed dormancy. The increased pollutant levels in the terrestrial domain by municipal sewage and extensive farm fertilization have led to the vulnerable quality of ground water by leaching of pollutants like heavy metals (zinc, cadmium, lead), chemical pesticides, fertilizers, and other micropollutants [33]. Nutrient secretion in land areas can lead to water nutrient enrichment through processes like eutrophication. Recent instances show the increasing risk of forest fires in zones contaminated with radioactive pollutants [34]. Increased deforestation due to various pollutants has directly or indirectly affected the increasing soil erosion rates and has displaced sediments and nutrient-rich soil surface causing loose soil [35] and thus promoting the events of flash floods.

#### 3.1.3. Aerial

Air pollution is a threat to the whole human civilization and is the sixth leading cause of death in the world. Aerial pollutants like carbon dioxide, fuel soot particles, methane, halocarbons, ozone, and nitrous oxides have contributed to causing one of the biggest environmental challenges, i.e., global warming [36]. Global warming leads to ocean acidity, increased sea levels due to melting glaciers and snow, enhanced tropical calamities such as storms, loss of viable agricultural grounds and animal habitats, and dysregulated water supply [37]. Some pollutants like nitrogenous and sulfur oxides produced by combustion interact with the atmosphere and result in acid deposition in the form of rain, fog, or snow. It is known to destroy the stability of the ecosystem, threaten food reserves, and can be speculated to cause a socioeconomic implication at a larger level socioeconomic implication [38]. The levels of air pollutants are increasing substantially in urban and periurban areas in developing countries like India. They in turn have affected the adjacent agricultural areas and have caused a threat to the crop production rates [39]. High particulate matter concentration can cause emission changes and physical, chemical changes in the atmosphere. The emissions of volatile organic compounds will be altered in the near future, which may further impact secondary organic aerosols [40].

### 3.2. Health Consequences

Air pollutants like oxides, particulate matter, and ground-level ozone are one of the most common health threats. Oxides such as CO (carbon monoxide) decrease oxygen delivery to body organs, impair vision, and at extreme concentrations are fatal. Sulfur oxides have been reported to be carcinogenic in nature if exposed for a long duration [41]. These pollutants have contributed significantly to problems such as cough, increased difficulty in breathing /decreased lung function, irritation in respiratory pathways, triggering asthma, and inflammation of bronchi and bronchioles. Besides adults, children also have been adversely affected; there have been increased cases of infant mortality, perinatal effects, allergy, cardiovascular and mental disorders, endothelial dysfunction, among them [42].

*Traffic-Related Air Pollution.* A mixture class of air pollutants can contribute to the generation of reactive oxygen species, creating an imbalance in free radical concentration that causes oxidative stress and initiates a risk of cancer development [43]. Increased oxidative stress and poor cardiovascular circulation cause inflammation in organs, which can cause mental health problems. Nitrogenous oxides and particulate fine and ultrafine matter have the capability to cross the blood-brain barrier and reach the brain, thus modulating Vaso regulation and even triggering neural inflammation and thus being a stepping stone for future neural dysregulations [44].

New pollutants are emerging and entering aquatic bodies: pesticides, chemically synthesized fertilizers, compounds such as dyes, detergents, and heavy metals have the potential to affect human health [45]. Eutrophic waters can cause skin-eye irritation, gastric inflammation, and vomiting in individuals. If high nitrogen levels are persistent, they may be a risk to infants <6 months of age, causing Blue Baby syndrome (decrease in the oxygen-carrying capacity of hemoglobin) [46]. Major pollutants even observed in drinking water, such as heavy metals—arsenic, lead, mercury, phthalates—have endocrine-disrupting properties. They can mimic, block, or interfere with the action of hormones present in the human body producing neurological or reproductive adverse developments. PFAS (Per- and polyfluoroalkyl substances), another type of pollutant, has been linked with infertility, thyroid dysfunction, cancer, and obesity [47]. Contaminants are known to cause problems like diarrhea, cholera, typhoid, malaria, and other congenital problems [48].

## 4. Traditional Methods of Removing Pollutants and Their Side Effects

Traditional methods like coagulation, adsorption, and advanced oxidation processes combined with electrochemical and biological processes (Table 1) have been used before the revealment of nanobiotechnology.

Coagulation and flocculation are traditional methods for the treatment of polluted water. They are used as a pretreatment of biological treatment, improving the biodegradability of waste and removing macropollutants [58]. Compounds like ferric chloride and polymers are added which destabilize the colloidal materials and small particles to agglomerate in large flocs that are settleable. It is a physiochemical approach to treat metal pollutants in industrial wastewater and remove soluble compounds by forming flocs [59]. It also aids in the elimination of suspended solids (SS) and organic material. Coagulation accompanied by ozonation has been used for waste sanitary landfill leachates [60]. The coagulation-flocculation process is also known to remove nutrient pollutants like P and nitrogen [61]. In comparison to this, adsorption is a surface phenomenon of organic as well as inorganic pollutant removal. It can be achieved through its subtypes like membrane adsorption where the contaminants are adsorbed to the membranes while the pollutant carrying source flows through it. Chromium(VI), zinc (II), and lead (II) are known to be successfully removed by polymer membrane-based adsorption methods and membrane-based adsorption methods, especially polypyrrole [62]. Activated carbon adsorption, as listed by the USEPA (Environmental Protection Agency), is one of the best control technologies for the removal of organic dyes and discharges from pharmaceutical companies [63]. Adsorption processes involving activated carbon have long been regarded as conventional treatment processes which can remove a multitude of pollutants [64]. *Zeolite Adsorption.* Natural zeolites known to exhibit high selectivity for heavy metals (cadmium, copper, zinc) and high abundance as compared to other chemicals synthesized for pollutant removal [64] have a high surface, large pores, thermally stable, cheap, chemically inert, and easily modified surface functional group that enables it to have excellent selectivity against pollutants [50]. *Biosorption*. Adsorbents are either prepared naturally or engineered artificially, giving them several advantages such as low cost, abundant raw material, high selectivity with regenerative ability, and performance comparable to conventional techniques. It can be used for the treatment of secondary and tertiary effluent pollutants [65].

Complete degradation of organic pollutants with mineralization can be achieved by advanced oxidation processes. Processes like ozonation, Fenton reaction, UV irradiation, peroxide handling, and their combination are being included in it. Polyphenols can be treated by Fenton and solar photo- Fentons. Photo-Fenton can also treat reoccurring organic compounds. AOP can aid in chemical oxygen demand and poly phenol removals up to 90 percent [66]. Pollutants such as toxic cyanide can be removed using a combination of NaClO and hydrogen peroxide forming singlet oxygen and hydroxide ions [67]. AOPs tend to produce highly biodegradable and less toxic products [54]. Photolysis, i.e., degradation by light (photons) combined with hydrogen peroxide, can be used for the degradation of pesticides with the assistance of solvents like trichloromethane and carbon tetrachloride [68, 69].

Heterogenous photocatalysis by semiconductors such as titanium dioxide, zinc oxide, ferric oxide, copper sulfide, and cadmium sulfide can be efficient in degrading the pollutants into biodegradable compounds with a less toxic footprint as well as inorganic and halide ions [51], with additional benefits of low cost, easily crystallizable forms, phytochemical stable nature, and nontoxic behavior [55]. Ozonation based on the oxidation and/or degradation of pesticides (belonging to the classes organochlorine, organophosphorus, and triazine) allows for high reductions in chemical oxygen demand and significant removal of aromatic compounds [70]. Electrochemical processes have been also used as traditional methods for pollutant removal. Electrocoagulation uses a metal sacrificial anode which doses water. It has advantages in providing cations without increasing the saline nature of the solution, is easily operatable, has rapid sedimentation rates, reduced sludge production, and has less amount of coagulant ions required for treatment [56, 71]. Electrochemical Advanced Oxidation Processes have enabled the removal of pollutants and their complete mineralization with an environment-friendly approach. Extensive hydroxyl ions are produced even under optimal conditions of current and catalyst [72]. The current usage promises complete automation of the process on a large scale [49]. Electrokinetic remediation is a potential technique used for the separation and remediation of areas contaminated with organic and inorganic pollutants, for clean-up of low permeable matrices. It uses mechanisms like electromigration, electroosmosis, and electrophoresis combined with or without electrolysis [53, 73]. Electro Fenton is an electrochemical-based oxidation method [57]. The oxidation of organic compounds occurs via hydroxyl radical generated by electrochemical oxidation indirectly, and both hydrogen peroxide and ferrous ions can be electrogenerated under in situ conditions [74]. Electrochemical oxidation is a widely used popular technique for removing organic pollutants [75]. The oxidation is direct or indirect [76]. However, the direct oxidation process is in general not in use due to its ineffective nature in the degradation of organic pollutants, but its powerful oxidating agents produced could be used in indirect oxidation processes [57]. Biological methods have been adopted for pollutant removal due to their low cost and green nature [52]. They are widespread and conventional in nature, cost-effective, and well studied and can be modified according to the local needs [49]. They can be combined with other processes like wet air oxidation, ozonation for their increased efficiency, and better removal of pollutants that are toxic and refractory in nature [77].

## 5. Role of Nanotechnology and Nanomaterials for Remediation of Pollutants

Nanotechnology is a branch of science and engineering that is creating “materials” on the scale of atoms and molecules. Conventional principles of physical and chemical sciences do not apply to these sizes. The appearance, toughness, conductivity, and sensitivity of materials change drastically between the nano- and macroscales. For instance, carbon “nanotubes” are hundredfold stronger than steel, as well as sixfold lighter [78]. Various types of nanoparticles, their characterization mechanism, and how they can be applied in various sectors ranging from the health industry to the removal of environmental pollutants have been elucidated in this review.

For the elimination of environmental pollutants, the environmental remediation process employs a variety of methods (e.g., surface assimilation, absorption, synthetic reactions, catalysis using light, and filtering) [79]. Nanomaterials possess a high ratio of their surface-area-to-volume, frequently resulting in greater biogeological and chemical reactivity compared to other macroscale materials. Their improved characteristics and efficacy make them particularly appropriate for carrying out such operations [80]. Nanoscale materials are being used in a wide array of fields, including science, the environment, industry, and medicine.

Over the last few years, an increase in the number of nanoscale products with environmental remedial applications has been created and deployed. Nanomaterials, for example, have been utilized to clean up contaminated soil and groundwater at various hazardous waste sites like those damaged by chlorinated solvents or in oil spills. Manufactured nanoparticles have physicochemical, surface, and optical-electronic characteristics that answer issues that were previously difficult to tackle with traditional methods. It can design unique ways for creating new methodologies, replacing current tools, and producing new materials and chemicals with high performance and low energy usage [81].

## 6. Different Methods for Nanoparticle Synthesis

**Figure 2:**
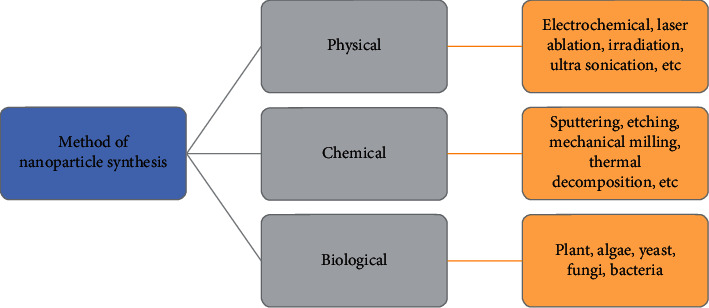
Method of nanoparticle synthesis.

Science and innovations have made rapid advances in the production of nanomaterials to obtain unique characteristics that differ from those of bulk materials. Nanoparticles can be prepared by chemical, physical, and biological methods (Figure 2).

### 6.1. Chemical Methods

#### 6.1.1. Using Polyols for Synthesis of Nanoparticles

Polyols have numerous hydroxyl groups attached to any organic compound. Nonaqueous liquid (in which the solvent is any other than water),falling under polyols is used as a solvent and reducing agent in this technique. The use of nonaqueous solvents in this technique has the benefit of reducing surface oxidation and assemblage [82]. If the synthesis is carried out at a somewhat elevated temperature with precise particle growth control, the polyol process can be used as a sol-gel technique in the production of oxide [83]. Oxide-based nanoparticles of vanadium, yttrium, manganese, cobalt, tin, lead, and titanium can be produced by this method. Because of its unique properties of high reducing ability and high boiling point, ethylene glycol is the most broadly adopted solvent in the polyol technique in metal oxide nanoparticles manufacturing [84]. Bimetallic alloys and core-shell nanoparticles can also be produced by this method [85].

#### 6.1.2. Reduction in Solution (Colloidal Method)

The use of reducing agents such as Na_3_C_6_H_5_O_7_ is one of the most essential ways. Turkevich suggested this method for the first time for the manufacture of Au monodisperse colloidal solutions in 1951. This approach allows for the creation of spherical nanoparticles. Later, the same approach was utilized to make silver nanoparticles, and the spread of these particles was bigger, ranging from 60 to 200 nm. In this method, a metal salt gives nanoparticles as residues upon reduction in a liquid solution. This method is generally used for the synthesis of gold, iron, molybdenum, and silver nanoparticles. For gold, a solution of chloroauric acid is heated to a boil. Trisodium citrate is used as a reducing agent. After mixing and stirring for 10 mins, seed crystals changed to gold nanoparticles.

#### 6.1.3. Electrochemical Synthesis

The capacity to precisely tune the chosen potential and the rejection of the potentially wasteful alternative half-reaction are the key advantages of this technology over a conventional chemical reaction [86]. The electrochemical production of Ag nanoparticles has received a lot of attention lately. The electrochemical process employed consisted of dissolving a metallic anode in an aprotic liquid [87]. Adding electrons to oxidize the positive electrode of the cell for solvable silver ions in acetonitrile containing tetra butyl ammonium yields Ag nanoparticles ranging in size from 2 to 7 nm. By adjusting the current density, the particle size can be determined. The electrochemical production of styrene-stabilized red luminous Si nanoparticles is also commercialized. Under UV stimulation, Si nanoparticles glow, which is useful in optics [88]. Platinum nanoparticles can also be synthesized by the electrochemical method. The desired Pt nanoparticles are deposited on the surface of the electrode with a size of about 10 nm [89].

### 6.2. Physical Methods

#### 6.2.1. Plasma Method

Another approach for producing nanoparticles is the plasma process. Radiofrequency (RF) heating coils create the plasma. The first metal is placed in a pestle, which is then placed in an evacuated chamber. High-voltage RF coils are wrapped around the evacuated chamber; then, the metal is heated above its evaporation point [90]. Helium is the gas employed in the technique, which after flowing into the system generates a high-temperature plasma in the region of the coils. Metal vapor forms on helium gas atoms and diffuses up to a cold collecting rod, where nanoparticles are collected and passivated by oxygen gas [86].

#### 6.2.2. Microwave Irradiation

There is the usage of microwaves to heat samples, which reduces reaction time and requires fewer materials. Furthermore, the use of microwaves allows for better control of reaction processes. The higher the effectiveness of nanoparticles with greater applicability, whether as ferrofluid, cell separation, or pollution removal, the closer they are to the spherical form and the more homogeneity between the forms [91]. Microwave-assisted irradiation can be used to make ZnS nanoparticles. XRD, SEM, and UV-Vis spectroscopy were used to analyze the generated ZnS nanoparticles. According to the XRD spectrum, the average size of the nanocrystallites was measured using the Debye-Scherrer formula, and they were determined to be around 6 nm [84].

#### 6.2.3. Radiation Dependent

Gamma radiation is repeatable, can control particle shape, yields monodisperse metallic nanoparticles, is simple, is inexpensive, and uses fewer toxins as a precursor: it uses the fewest reagents, uses a reaction temperature close to room temperature with as few synthetic steps as possible, and minimizes the amount of generated by-products and waste [92]. The radiolytic reduction has been shown to be an effective method for producing monosized and widely distributed metallic clusters [93]. The excitation and ionization of the solvent are the major consequences of high-energy gamma rays interacting with a solution of metal ions [94].

### 6.3. Biological Method

Green synthesis is necessary to avoid the creation of undesired or hazardous by-products by developing dependable, long-term, and environmentally friendly manufacturing processes. To attain this aim, suitable solvent systems and natural resources are required. To accommodate diverse biological components, green production of metallic nanoparticles is being used.

#### 6.3.1. Plant

Plant leaf extracts contain important elements to be used in the process of synthesis of nanoparticles. At various experimental parameters, the plant extract is combined with metal precursor solutions [95]. The parameters that determine the settings of plant leaf extracts are acknowledged to affect the pace of nanoparticle production, as well as the output and stability of the particles [96]. Flavones, terpenoids, sugars, ketones, aldehydes, carboxylic acids, and amides are the major phytochemicals involved in nanoparticle bioreduction [97]. The enol-to-keto-transformation regulates the generation of biogenic Ag nanoparticles in sweet basil extracts [98]. Sugars found in plant extracts, such as glucose and fructose, also have a role in the creation of metallic nanoparticles [99]. Plant extracts are biomolecules composed of carbohydrates and proteins that function as a reducing agent in the synthesis of metallic nanoparticles [100]. Geranium leaf extract contains terpenoids that actively participate in the conversion of Ag ions into nanoparticles [101]. Eugenol is a major terpenoid found in cinnamon extracts and is required for the bioreduction of the metal ions HAuCl_4_ and AgNO_3_ [102].

#### 6.3.2. Bacteria

Commercial biotechnological applications such as environmental remediation, genetic manipulation, and biosorption have all made use of bacterial species [103]. Bacteria have the ability to decrease metal ions and are important candidates in the creation of nanoparticles [104]. Due to its economic potential and viability, green synthesis technology delivers a clean, nontoxic, and environmentally acceptable technique for the production of metallic nanoparticles [105]. Bacteria like *Delftia acidovorans* can synthesize pure Au nanoparticles [106]. Delftibactin is considered a nonribosomal peptide (NRP) that has been linked to the production of Au nanoparticles due to its ability to induce resistance to toxic Au ions. Due to the formation of inert gold nanoparticles bound to delftibactin, the transition metal gold had no toxicity toward bacteria [107]. *Bacillus licheniformis* is said to have produced Ag nanoparticles within its cells [108].

#### 6.3.3. Fungi

Green synthesis of metal/metal-oxide nanomaterials by fungi is also a very rapid method. Because they include a range of enzymes, they are better green means for the synthesis of metal-based nanoparticles [109]. In comparison to bacteria, the competent fungus can generate greater volumes of nanoparticles [110]. *Trichoderma reesei* has an advantage over other fungi in that it is a well-studied organism that can be controlled to produce a large number of enzymes and may aid in increasing the rate of nanoparticle synthesis [111]. The fungus *Neurospora crassa* can be used in the biological manufacture of platinum nanoparticles. Single PtNPs with diameters ranging from 4 to 35 nm were generated intracellularly [112].

#### 6.3.4. Algae

Heavy metals are thought to accumulate in algae, which could be used in the biogenic manufacture of metallic nanoparticles [113]. The dried unicellular alga *Chlorella vulgaris* could produce tetrahedral, decahedral, and icosahedral nanoparticles that aggregated near the surface. The proteins in the algal extract serve as a stabilizing agent, a reducing agent, and a shape-control modifier, among other things [114]. *Sargassum wightii*, a marine alga, has the ability to produce extracellular Ag and Au nanoparticles [115]. The gold nanoparticles created are of real yield since they are made from living *Euglena gracilis* microalga cells that have been cultivated under either mixotrophic or autotrophic conditions [116].

## 7. Types of Nanoparticles and Their Characterization Methods

Nanoparticles can be divided further into many subtypes according to broader categories like material type, dimensions, or 3D structure and origin.

### 7.1. Basis of Origin

Nanoparticles can be either natural in origin or engineering (synthetic). Natural nanoparticles are produced in nature by biological processes and organisms or induced by human activity [117]. Synthetic or engineered nanoparticles as their name describes are nanomaterials produced by mechanical processes, exhaustive mechanisms, or chemical, biological, and physical processes individually or in combination [118].

### 7.2. Basis of 3D Structure

On the basis of structure, they can be classified into three categories: one dimension, two dimensions, and three dimensions. In zero D, the electrons cannot move in any axis; i.e., they are dimensionless; in 1 D, they can move along a single axis followed on by 2 D and 3 D where they can move in two and three axes, respectively, which is irrespective of the axis combination [119]. One-dimension nanoparticles are thin films of sizes ranging from 1 to 100 nm. Two-dimension nanoparticles generally include nanotubes, e.g., carbon nanotubes. Three-dimension nanoparticles consist of dendrimers, quantum dots, and fullerenes (like carbon 60) [120].

### 7.3. Based on Material Type

Nanomaterials can be subclassified according to the material they are made of such as inorganic, organic, carbon-containing, or composite substances. Inorganic-based nanomaterials are made of metals or their oxides, e.g., gold, silver [121], titanium dioxide, zinc oxide, or silicon-based compounds, etc. [119]. Organic nanomaterials are obtained from organic matter excluding carbon-based or inorganic-based sources; they are present in forms like micelles, liposomes, and polymeric particles. Composite nanoparticles are multiphasic in nature such that one of their phases can combine with other similar or dissimilar nanoparticles in bulk, complex formations, e.g., a gold nanoparticle in composition with ceramic or a polymer. Carbon-based nanoparticles consist of fullerenes, nanotubes, and nanofibers found in generally two-dimensional morphological structures [122–124].

### 7.4. Characterization

**Figure 3:**
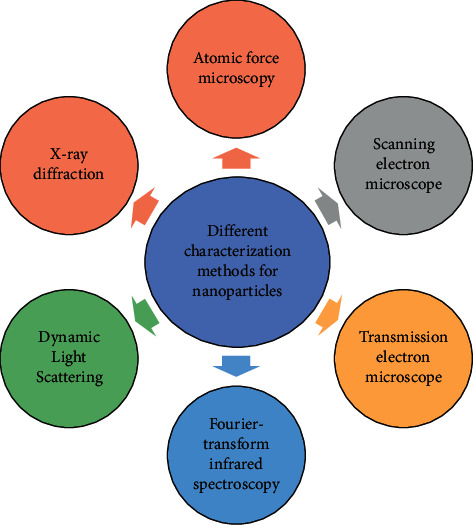
Different characterization methods for nanoparticles.

Various techniques (Figure 3) have been used for characterization, including UV-visible spectroscopy, that is, based on the principle of surface plaque resonance (SPR) [125]. Another technique is X-ray diffraction (XRD) to determine the crystalline phase [126–128]. Fourier transform infrared (FTIR) spectroscopy helps to identify the functional groups present in the sample. Characterization of nanoparticles based on size, morphology, and surface charge was performed using atomic force microscopy (AFM), scanning electron microscopy, and transmission electron microscopy [129–131]. Dynamic light scattering is used to measure nanoparticles size.

## 8. Different Application of Nanoparticles

The usage of nanoparticles/materials has shown tremendous growth worldwide from medical grafts to sensing tools. They have revolutionized how various techniques happen. Green synthesized particles especially silver nanoparticles have antibiotic activities; they have been used for medical implantations as silicone artificial heart valves, catheters, bone prosthetics, dental sets, and bandages [132]. The key ability of particles at the nanoscale has promoted the formation of biosensors for the easier diagnosis of diseases, checking the progression of the problem, as well as tracking the progress of the given therapy [133]. Green synthesized biodegradable nanoscale particles can have a sustained drug release for a long period of time without reporting the risk of infections [134]. Many studies have reported the multifactorial antimicrobial property of green nanoparticles. Generally, nanoparticles either function extracellularly or intracellularly to alter the bacterial growth rates such as inhibition of cytological transport and signaling [135]. Antibiotic activities of NP antifungal properties have also been reported. These particles can degrade cell walls and have been found to be as effective as 87% in studies with concentrations as low as 6 mg/ml [136].

Through the usage of gold nanoclusters and in vivo studies, carcinogenic cells were bioimaged with the help of fluorescent objects in cell lines such as hepatocarcinoma cell lines and leukemia cell lines. These nanoparticles are in clustered form with fluorescence activity [137]. Huang et al. created nanoparticles of carbon which were negligibly cytotoxic and highly biocompatible with hydrophilic nature and compatible nature. The blue photoluminescence cells were used to stain human HeLa cells and green fluoresced imaging [138]. Through recent advances in technology, antineoplastic traits of synthesized nanoparticles, which can have much lower side effects, have been the topic of research. These nanoparticles are very effective, provide a small amount of negative impact and target tumorigenic cell lines, and can also be used in combination with high-dose drugs to achieve the correct therapy [139]. Target therapies with a combination of anticarcinogenic nanomaterials use carriers like polymeric micelles, dendrimers, and liposomes. Inorganic metallic NM including metals and metal oxides is a promising approach for the future in cell imaging, using it as sensors, and drug delivery [140].

## 9. Nanomaterials in Remediation of Soil

Soil contamination and degradation continue to be a significant environmental problem, with remediation becoming a worldwide challenge. Degrading soil reservoirs throughout the world have a great influence on agricultural production and food safety; therefore, this needs to be addressed right away [141]. Heavy metals (remains that become toxins), herbicides, and POPs-persistent organic pollutants contaminate soil, adding to the issue. Because of biomagnification, soil contaminated with these chemicals provides an increased chance of intoxication of the food chain. Both the urgent production to feed more people and the difficulty of avoiding additional land deterioration have a significant impact on agricultural production [141]. The immobilization or adsorption procedure is one of the most commonly utilized treatment techniques. The effectiveness, low cost, and environmentally favorable approach for removing metal pollutants from soils are some of the most prominent benefits connected with this sort of remediation technology [142]. Carbon-based nanomaterials, for example, carbon nanotubes, oxides of metal (ferric oxide and titanium oxide), and various nanocomposites are some of the engineered nanomaterials that have been utilized to immobilize soil pollutants [143]. For example, ferric oxide nanoparticles have an extraordinary potential to absorb and immobilize heavy metals such as Cd and As from various media samples [144]. Oily sewage is an adverse problem affecting the aquatic local life [145]. Iron nanoparticles are successful in removal of Total Petroleum Hydrocarbons (TPHs) from water with enhanced results of 88.34% [146]. Thus, nanotechnology-based treatments are able to provide high-performance treated water which has less impurities and toxic substances combined with the removal of heavy metals. Nanoscience's can be combined with biological treatments for reducing the hazards of environmental substances. Organic dyes can be efficiently decolorized through industrial mechanisms backboned by nanoparticles [147]. Gold and silver nanoparticles are very successful catalyst in dye degradation, the basic process being two stepped; first the accumulation of electrons on particle surface and second the diffusion of dye for a reduction reaction [148].

Magnetic nanoparticles have an extended range of use within adsorption and catalytic pollution treatment [149]. Polyacrylamide-modified magnetite nanomaterials can be used, as they provide soil erosion and arsenate leaching [150]. On-site immobilization of pollutants has received a lot of attention as a feasible and economical method of remediating polluted soils. Various NM-based full form modifications have been studied, to find a relevant apt material that takes into account low cost, high efficiency, better stability, least harmful environmental effect, and maximum reusability. By reducing the exchangeable percentage of metals in polluted sediments and soils, nanohydroxyapatite particles successfully immobilized metal concentrations in pore water [151]. This research stated that utilizing sodium carboxymethyl cellulose-stabilized nZVI, they were able to remove 80 percent of soil-bound Cr (VI). Redox-sensitive elements as pollutants can be treated by abiotic reductive reactions directly impacting their mobility and toxicity in soil alone or in combination with immobilization techniques. The addition of nZVI to pyrene-adulterated soil, for example, led to strong reducing conditions, allowing pyrene to be removed through reduction. Nanomaterials are also used in advanced oxidation processes (AOPs) that employ various oxidants for the breakdown of organic contaminants in polluted soil [152]. Highly reactive radicals (water radical, sulfur dioxide radical) can be generated by combining hydrogen peroxide or persulfate with soluble Fe (II) for the efficient oxidative breakdown of organic contaminants. However, acidic pH (which is necessary to avoid Fe (II) precipitation) and related drawbacks, such as the expense of early acidification and negative impacts on soil quality and microorganisms, restrict these activities. As a result, solid iron phases have been proposed instead of soluble Fe (II) to allow chemical oxidation without pH alteration. Iron particles when used with chelating ligands act as an enhancement in the process [153]. Green nanoparticles have a very large scope for providing solutions against environmental pollutants and provide scope for the treatment of localized environments contaminated with metal ions toxic in nature. These materials can be useful in remediation and cleaning waste sites. Ferrous NP can be applied for disinfection of heavy metal contaminated water as well as heavy metal-containing soil, additionally being effective eco-friendly fertilizers. These nanofertilizers can synchronize plant nutrient uptake while decreasing groundwater contamination as they are immobilized and will not change into permanent chemical or gaseous forms that are unattainable for the growing plant [154]. Magnetite (Fe_3_O_4_) andgreigite (Fe_3_S_4_) with siliceous material produced using bacteria have been used in optical coatings for solar energy applications [155].

## 10. Nanomaterials in Remediation of Water Pollution

Wastewater discharge from commercial and industrial factories, as well as untreated residential sludge and synthetic contaminants influx to aquatic resources, is extremely harmful to humans as well as for the ecosystem. Heavy metal ions, organic compounds, and oils are the primary water pollutants in this regard, and they can render any water stream unfit for consumption [156]. Through the advancement of nanotechnology, a wide spectrum of novel nanomaterial-based technologies has emerged. Nanomaterials, as adsorbent systems, have a huge surface area for reactions at a very smaller relative weight, are manufactured at a lower price than activated carbon, and can effectively withdraw contaminants. A wide array of nanomaterial-based adsorbents in various shapes and parameters like dimensionalities have been studied in this respect, including zero-dimensional nanoparticles, one-dimensional nanofibers and nanotubes, two-dimensional nanosheets, and three-dimensional nanoflowers [157]. In terms of composition, there is a lot of variety, and various carbon-based and noncarbon-based nanomaterials can assist in cleansing aquatic bodies. The segregation process depends on the contaminant's chemical/physical interaction with the surface of the nanomaterial. Nanomaterials can also function as a major manufacturing component of the permeable segregator unit as a nanocomposite supreme filtration membrane, or as a thin film addition to enhancing the hydrophilicity and thermomechanical characteristics of the membrane [158]. Zinc and titanium oxides, ceramic, nanowire, and polymeric membranes, carbon nanotubes, and submicron particles are used in various remediation ways such as lysis, filtration, adsorption, and oxidation [159].

Dye pollutants are deadly because of their carcinogenic nature. Dyes have been used in textiles, paintings, pigments, and other products for thousands of years. Around 0.1 million different synthetic dyes are manufactured on an industrial level today. Talking about yearly usage, roughly 1.6 million tonnes of dyes are utilized. During usage, fifteen percent of the total is squandered [160]. Crocein orange G and acid green 25 adsorption capability is high in chitosan sugar-coated magnetite nanoparticles. A magnetic field, interestingly, could easily recover the adsorbent nanoparticles [161]. Fe_3_O_4_/activated carbon nanoparticles capable of separating 138 mg/g and 166.6 mg/g dyes (methylene blue and brilliant green) have been produced based on this idea [162]. In removing methylene blue dye from water, electrospun polyether sulfone nanofibers having vanadium nanoparticles are used. Because the nanofibers have a low isoelectric point, they develop a large, highly hydroxylated surface area in an alkaline medium, which promotes the adsorption of cationic methylene blue molecules [163]. Adsorption has the disadvantage of not being able to totally “eliminate” or “neutralize” dye contaminants. Instead, it collects and gathers dye constituents exclusively by moving them at different molecular phases. This functionality may be difficult to implement since the dye-based sewage is difficult to remove and is adsorbed on the rarely reused membrane [158]. A number of progressive oxidation processes that release hydroxyl radicals (O̊H) hold promise for the process of removing colors from textile effluents in this respect. Hydroxyl radical is highly reactive and oxidizes refractory organic contaminants due to its unpaired electrons. The increasing rate of photoreaction, which is photocatalysis, has become one of the most investigated advanced oxidative remediation methods and is regarded as a realistic destructive method for dyes (organic) and pesticides due to less cost. Metal oxides that have properties of semiconductors such as zinc oxide and titanium oxide demonstrated impressive photodecomposition capability when used to degrade dye contaminants [164]. Despite semiconductors' significant potential for decomposition in presence of the light of different dyes (organic) contaminants, industries with affiliated visible light photocatalysts have low steadiness or efficiency when exposed to light semiconductors having their energy gaps falling in the visible light spectrum that belongs to groups II–VI that have been offered as majestic, feasible options to address such issues [165].

## 11. Nanomaterials in Remediation of Air Pollution

Nanotechnology can help to clean up the air in a variety of ways. For gaseous reactions, one method is to use nanocatalysts with a larger surface area. Catalysts accelerate chemical processes that convert hazardous vapors from automobiles and industrial facilities into innocuous gases. The removal of air waste to the ground is ineffective; therefore, the filtered chemicals posed a difficulty for disposal [166]. Filters are beneficial in the case of air pollution. A filter has a small porous construction that allows gas to flow through while particles stay trapped inside. The membrane of the filter treats toxins using three mechanisms: filter structure surface contact, inertial force applied while moving the direction of gas, and electric charge generated by particle and filter construction [167]. The primary flaw of filters is that they cause a pressure drop, which necessitates a lot of energy usage. Nanofilters are more efficient than traditional filters, as their pores range from 1 to 10 nm, thereby efficiently removing a variety of microorganisms and organic pollutants. Carbon nanotube-based membranes have a better capability for separating CO_2_ from other gases [168]. Carbon nanotubes can capture gases at a pace that is 100-fold faster than current gas separation techniques. As a result, they are suitable for large-scale applications [169]. There seems to be a reverse connection between the efficiency of the gas separation process and the related volume flowing through traditional layers, but carbon-based nanotube membranes cover this. Nanomembrane technology may be utilized to separate and purify gases and pollutant vapors in a variety of sectors, as well as to prevent their discharge into the environment [170]. Nanosensors are a cutting-edge device which can identify and take action for a detectable physical change on such a nanometre scale. Nanoparticles, which are small 3D circular substances, are used as sensors and provide information when a toxin is present in a space [171]. One of the hazards in the commercial sector is the risk of obnoxious and hazardous spillage. Sensors used mostly at commercial levels have recently discovered small amounts of these gases. This requires the use of technical advancements in the creation of faster and more accurate sensors. This type of biosensor consists of single or multimembrane nanotubes that can capture dangerous gas molecules. Smart dust is a new kind of nanosensor. The ultimate goal is to develop a series of advanced sensors in the form of ultralight nanocomputers. Such silicon-based ultrafine particles have a structure that permits them to wirelessly transfer data collected to the main server. The crucial data transportation rate with this sort of sensor is about 1 kbps or more. These solar-powered nanosensors can easily stay hung in the air for hours. Smart dust can convey information such as climate conditions, humidity, and amount of pollutants for up to 20 kilometers and can provide conditions to continuously manage air pollution in a given area [171].

## 12. Conclusion and Future Prospects

Nanoparticles have revolutionized the future approach for litigation of environment with biotechnology. With the increased progress of humans, industrialization and consumption of reserves are present in nature. There is an urgent requirement for the removal of these pollutants with the least possible side effects to the Earth, though not lamenting the side effects of earlier existing processes like Fenton, coagulation, adsorption, advanced oxidation etc. and their combinations as well. A more sustainable, holistic approach to the tackling of this grave issue can be nanoparticles that may be active while working on both small- and large-scale levels. Diversified nanoparticles have become handy for removing specified pollutants in their native area like sewage spills, landmines, etc. Moreover, they cause a minimum toxic effect during production rates and thus can be well researched forward so that they are more compatible in the future with decreased production costs.

## Figures and Tables

**Table 1:** Side effects of using traditional processes for pollutant removal.

Pollutant removal method	Side effect	Reference
Biological processes	(i) Cannot treat toxic or refractory organic pollutants and have limitations.	[49]
Zeolite adsorption	(i) They might be highly hydrophilic on template removal because of their high surface silanol density, leading to low adsorption for hydrophobic pollutants.	[50]
Photocatalysis	(i) Economic constraints for the high level of mineralization. Postreaction products of photocatalysis could still remain toxic.	[51, 52]
	(ii) Photo corrosion is a typical drawback, postseparation inorganic catalysts. Semiconductor photocatalysts are unstable under light irradiation.	
Electro kinetics	(i) There might be ionic motion, crystallization of metal salts, and dehydration which prevented removal of inorganic pollutants.	[53]
Electrochemical advanced oxidation processes	(i) High cost, high energy consumption for complete mineralization of pollutant.	[49]
Advanced oxidation process	(i) Considerably affected by pollutant nature, type, and concentration of oxidants and catalyst, reactor configuration.	[54, 55]
	(ii) Release toxic and less biodegradable by-products in extreme cases.	
Electrocoagulation	(i) Myriad of designs for reactor formation.	[56]
Ozonation	(i) Difficult mineralization of pollutants due to the presence of hydroxyl ion scavengers.	[43]
Classical Fenton process	(i) Unsafe storage, transportation, and handling of hydrogen peroxide for large treatments.	[57]

## Data Availability

The data used to support the findings of this study are included within the article.
